# Characterization and phylogenetic analysis of the complete mitochondrial genome of prickly redfish *Thelenota ananas* (Jaeger, 1833)

**DOI:** 10.1080/23802359.2021.1920509

**Published:** 2021-07-06

**Authors:** Hongtao Liu, Minghui Shen

**Affiliations:** Hainan Provincial Key Laboratory of Tropical Maricultural Technologies, Hainan Academy of Ocean and Fisheries Sciences, Haikou, China

**Keywords:** *Thelenota ananas*, mitochondrial genome, phylogenetic analysis

## Abstract

In this study, the complete mitochondrial genome (mitogenome) of prickly redfish *Thelenota ananas* (Jaeger, 1833) was determined and characterized from the South China Sea using next-generation sequencing. Our results showed that the length of the whole mitogenome in prickly redfish was 15,858 bp and the mitogenome was consist of 22 tRNA genes, two rRNA genes, 13 protein-coding genes (PCGs), and one control region. Furthermore, the nucleotide composition was significantly biased (composition of A, G, T, and C was 28.20%, 22.64%, 33.53%, and 15.63%, respectively) with AT contents of 61.73%. All the PCGs shared a standard initiation codon ATG or GTG. *CYTB* and *ND6* genes terminated with an incomplete stop codon T, while others ended with TAA or TAG. Our phylogenetic analysis showed that *Thelenota ananas* was clustered with the species of genus *Stichopus,* and formed sister branches with species of other genera within the family Stichopodidae clade.

As a member of the Stichopodidae family, *Thelenota ananas* (Jaeger, 1833), has also been known as pineapple sea cucumber or prickly redfish. This species is a kind of sea cucumber characterized by the large size, warm colors, and pointed, star-shaped teats covering the entire body, grouping in rows of 2 or 3. The habitat of prickly redfish ranges throughout the Indo-Pacific, excluding Hawaii (Oury et al. 2019), distributing from South Africa to India (Conand 2008), Australia, Japan, and China. The prickly redfish has been always found along slopes and passes within reef zones. Generally, it is common in the water area of 10–20 m, but the deepest can extend to 35 m along the outer reef (Conand and Mangion 2002). Due to the medium-high value as food and as an aphrodisiac, prickly redfish has been exploited commercially, the populations have decreased drastically. Currently, the prickly redfish has been listed as Endangered in the International Union for Conservation of Nature (IUCN) Red List (Conand et al. 2014). Studies about the taxonomic revision of Stichopodidae have been one of the most controversial issues in recent years, the incongruences between morphology-based taxonomic systems and molecular systems have been debated for years (Zhong et al. 2019). Previous studies showed that the mitochondrial genome is an excellent molecular marker for researches on phylogenetic relationships and species identification in animals. Here, we reported the complete mitochondrial genome sequence of *T. ananas*, providing a better understanding of higher-level relationships within Stichopodidae.

Specimens of the prickly redfish in this study were obtained from the Xisha Islands, Sansha, China (N16°50′24.37″, E112°21′17.70″), and deposited in the Hainan Marine Science and Technology Museum (Hongtao Liu, xmulht@gmail.com) under the voucher number E20201203TA in Qionghai research base of Hainan Academy of Ocean and Fisheries Sciences. The libraries with an average length of 350 bp were constructed using the NexteraXT DNA Library Preparation Kit, and sequencing was performed on the Illumina Novaseq platform (Total Genomics Solution Limited, SZHT) the 150 bp average length of the generated reads. The complete mitochondrial genome of prickly redfish was assembled with 3.81 G clean reads using the de novo assembler SPAdes 3.11.0 (Bankevich et al. 2012) and annotated using the MITOS (http://mitos.bioinf.uni-leipzig.de/index.py). Based on the available nucleotide sequences of mitogenome in 14 species in the GenBank, the phylogenetic analysis was carried out using IQ-TREE v1.6.12 (Nguyen et al. 2015) with maximum likelihood (ML) method, the bootstrap replicate was set as 1000, and the best-fit model was chosen mtMet + F+R5 according to Bayesian information criterion (BIC).

The whole mitogenome of the prickly redfish (GenBank Accession No. MW548268) is 15,858 bp in length. The overall base composition was 28.20% A, 22.64% G, 33.53% T, and 15.63% C respectively. The 61.73% of (A + T) underlined the great preference for AT in the prickly redfish mitogenome. The mitogenome we characterized here consists of 22 tRNA genes, two rRNA genes, 13 protein-coding genes (PCGs), and one control region. The *ND6* gene, five tRNA genes, and one control region were located on the light strand, the others were encoded by the heavy strand.

The sequencing of the mitogenome in the prickly redfish showed that 22 tRNA genes vary in length from 65 bp to 73 bp. tRNA-Leu and tRNA-Ser both have two type copies respectively in the mitogenome. The 12S rRNA was 837 bp long and located between tRNA-Glu and tRNA-Phe. The 16S rRNA was 1450 bp long and located between *COX1* and *ND2*. The control region is 260 bp, located between tRNA-Pro and tRNA-Thr. There were 11 overlapping regions of 1–7 bp in length. The longest overlapping region was located between *ND4* and *ND4L*. The mitochondrial genome was consist of 14 intergenic sequences varying from 1 to 740 bp in length (Supply Table S1). All the 13 PCGs shared a normal initiation codon ATN or GTG. Simultaneously, 11 PCGs terminated with the common stop codon TAA or TAG, besides *CYTB* and *ND6* genes ended with an incomplete stop codon T.

The phylogenetic tree (Figure 1) showed that *T. ananas* was clustered with the species of genus *Stichopus* at first place, then formed sister branches with species of other genera within the Stichopodidae family clade with high bootstrap value. Our results were similar to previous studies based on *COI* and 16S mitochondrial DNA (Byrne et al. 2010). Taken together, the newly sequenced mitochondrial genome of *T. ananas* characterized here should contribute to a better understanding of phylogenetic relationships of sea cucumber species in the Aspidochirotida, and the molecular identification, population genetic and evolutionary biological studies of the prickly redfish *T. ananas*.

**Figure 1.**
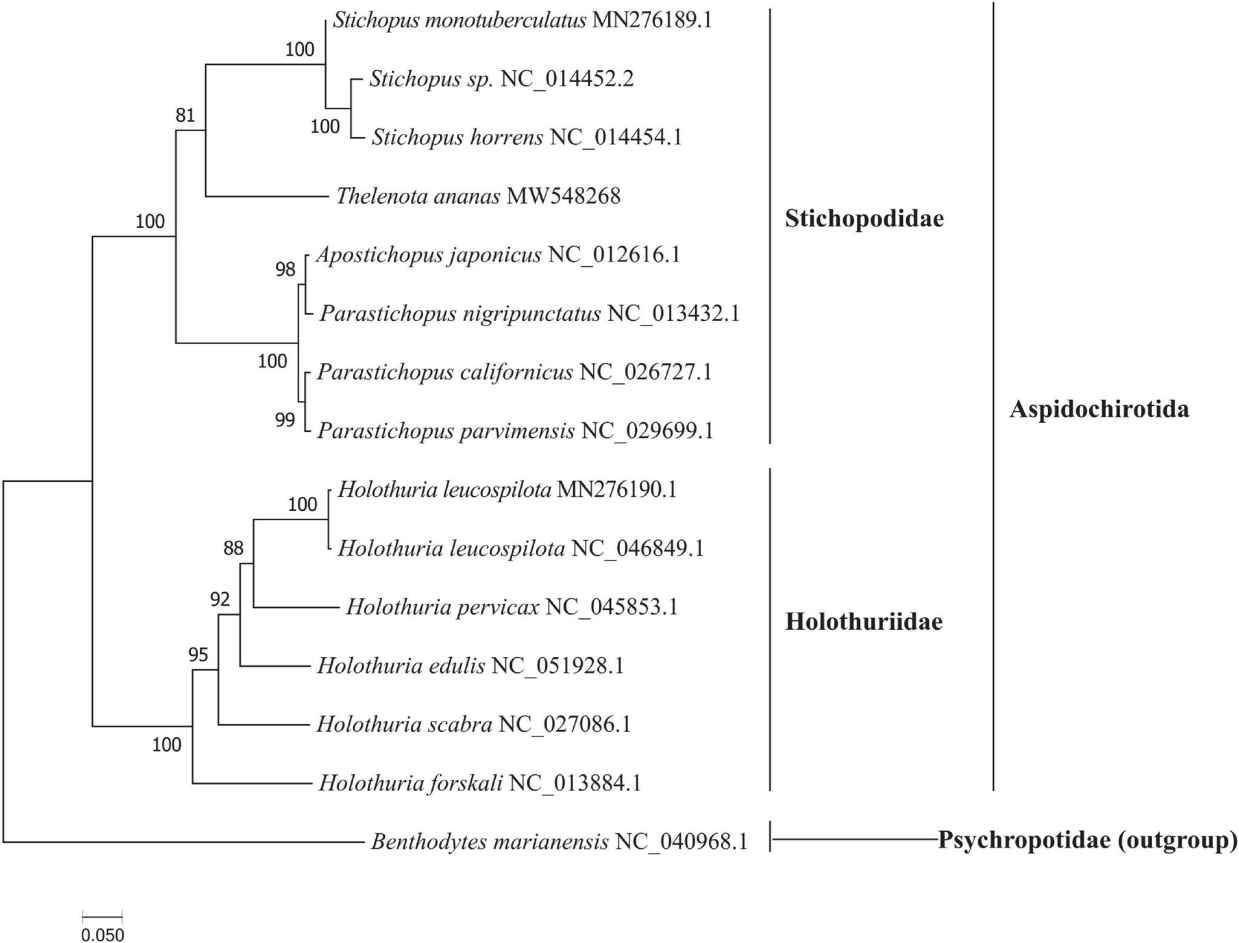
The maximum likelihood tree of *T. ananas* and 14 other species based on the 13 PCGs. The GenBank accession number for each species is indicated after the scientific name. The ML tree was carried out using IQ-TREE v1.6.12 with 1000 bootstrap replicates, the best-fit model is mtMet + F+R5. The bootstrap values were labeled at each branch node. *Benthodytes marianensis* was used as outgroups.

## Data Availability

The genome sequence data that support the findings of this study are openly available in GenBank of NCBI at (https://www.ncbi.nlm.nih.gov/) under the accession no. MW548268. The associated BioProject, SRA, and Bio-Sample numbers are PRJNA698270, SRR13590426, and SAMN17711066 respectively.
